# Distinct alterations of fecal microbiota refer to the efficacy of adalimumab in Crohn’s disease

**DOI:** 10.3389/fphar.2022.913720

**Published:** 2022-08-11

**Authors:** Liang Chen, Zhanjun Lu, Dengfeng Kang, Zhongsheng Feng, Gengfeng Li, Mingming Sun, Zhanju Liu, Wei Wu, Leilei Fang

**Affiliations:** ^1^ Center for Inflammatory Bowel Disease Research, The Shanghai 10th People’s Hospital, School of Medicine, Tongji University, Shanghai, China; ^2^ Department of Gastroenterology, Shanghai General Hospital, School of Medicine, Shanghai Jiao Tong University, Shanghai, China

**Keywords:** adalimumab, mucosal healing, fecal microbiota, biomarker, Crohn’s disease

## Abstract

**Background and Aims:** Anti-tumor necrosis factor mAb (i.e., adalimumab, ADA) is currently used in the treatment of patients with Crohn’s disease (CD). However, its regulation on fecal microbiota is still not fully understood.

**Methods:** A retrospective analysis was conducted on 115 patients with CD who received treatment with ADA for 12 weeks at the Inflammatory Bowel Disease Center in Shanghai Tenth People’s Hospital and Department of Gastroenterology in Shanghai General Hospital. The Crohn’s disease activity index (CDAI) evaluation was applied to patients before ADA therapy at week 0, 4, 8, and 12. Clinical remission (CR) was defined as the CDAI < 150. All patients underwent ileocolonoscopy or enteroscopy at baseline (week 0) and week 12. Crohn’s Disease Endoscopic Index of Severity (CDEIS) scores were calculated by two experienced physicians to assess endoscopic activity. Mucosal healing (MH) was assigned a CDEIS score between 0 and 3. Fecal samples were collected from eight CD patients at baseline and week 12, and the microbiota was analyzed by using 16S RNA sequencing.

**Results:** At week 12, CR was achieved in 70.6% (72/102) of the patients with active CD. A total of 47.1% (48/102) of the patients with active CD attained MH, among which, 56.6% (30/53) of the patients with mildly active CD (3 ≤ CDEIS <9) and 48.0% (12/25) of the moderately active CD patients (9 ≤ CDEIS <12) attained MH, but only 25.0% (6/24) achieved MH in severely active CD patients (CDEIS ≥12). The efficacy of ADA was not associated with lesion locations (*χ* ^2^ = 0.409, *p* = 0.815). Unexpectedly, we found an increase in protective microbiota at the genus level (e.g., *Barnesiella*, *Anaerostipes*, *Tyzzerella*, *Lachnoclostridium*, and *Lachnospiraceae_unclassified*) but a decrease in pathogenic bacteria (*Escherichia–Shigella*) in fecal samples of the ADA-responsive group (ADA-R) when compared with those in the ADA-nonresponsive group (ADA-NR). Notably, the gene *bglX* coding β-glucosidase and *gph* encoding phosphoglycolate phosphatase were enriched in fecal samples of ADA-R. Conversely, the abundance of genes coding ATP-binding cassette (ABC) transporter system proteins was significantly enriched in fecal samples of ADA-NR when compared with that of the ADA-R.

**Conclusion:** This study reveals that ADA markedly improves clinical remission and induces MH in mildly to moderately active CD patients and that distinct changes in the gut microbiota can be used to predict the efficacy of ADA.

## Introduction

Crohn’s disease, a kind of inflammatory bowel disease (IBD), is thought to be caused by an intricate interaction between microbiota and the intestine in genetically susceptible individuals. Trillions of bacteria harbored by the intestine are indispensable for maintaining intestinal homeostasis. The importance of microbiota for IBD has been established in various studies concerning fecal stream diversion and biotherapy, and in experimental mouse models (Rutgeerts et al., 1991; Sartor, 2004; Chen et al., 2019).

Anti-TNFs are monoclonal antibodies those neutralize soluble and transmembrane tumor necrosis factor (TNF), a pro-inflammatory cytokine predominantly involved in the inflammatory cascade of CD (Fang et al., 2018). The anti-TNFs used in clinical practice are infliximab (IFX) and adalimumab (ADA), which are for remission induction in patients with active CD. IFX has been approved for the treatment of patients with CD for years in various countries besides China. Nevertheless, up to 30% of CD patients show no clinical benefit following IFX induction and up to 50% discontinue it due to loss of response over time (Papamichael et al., 2019). Both these unwanted outcomes can be mostly explained by inadequate drug concentrations or the development of excessive antibodies to IFX. As a humanized monoclonal antibody, ADA has been shown to induce and maintain remission in global clinical trials of patients with CD (Hanauer et al., 2006; Jilani and Akobeng, 2007). In the Chinese program which is a 26-week, multicenter, phase III study to evaluate the efficacy and safety of ADA in Chinese CD population, patients with moderately to severely active CD who were receiving ADA achieved clinical remission in significantly greater proportions than those patients receiving placebo, and all enrolled patients were anti-ADA antibody-negative throughout the study (Chen et al., 2020). Although it has been widely used in other countries, ADA had not been introduced to Chinese CD patients until 1 January 2020. Thus, there is still a lack of real-world data on the efficacy and safety of ADA in Chinese CD patients.

The therapeutic aim for IBD has shifted from symptom control to mucosal healing. However, approximately one-quarter of CD patients are primary nonresponders to anti-TNF agents, and one-third of the responders experience a loss of response over time (Rajca et al., 2014). The reasons for treatment failure are, however, largely unknown. Therefore, it is essential to improve treatment effectiveness of anti-TNF agents, such that these patients would show clinical and endoscopic benefits after optimal response. In recent years, evidence of gut microbiota alterations have been found during IFX therapy. Rajca et al. (2014) observed a lower rate of *Firmicutes* in the gut of CD patients who relapsed after IFX withdrawal when compared with that of patients in long-term remission, through exploring changes in the gut microbiota. Research by Zhuang et al. (2020) reports that IFX therapy modified the gut microbiota structure in patients with active CD, and the alterations of *Lachnospiraceae* and *Blautia* may be potentially predictive biomarkers of clinical and endoscopic efficacy during IFX therapy at early stages. Previous studies have suggested that gut microbiota are important for disease monitoring and IFX treatment decision-making, whereas few studies have focused on the association of gut microbiota and ADA efficacy in treatment using ADA.

In this study, we evaluated the efficacy and safety of ADA in remission induction and maintenance in adult Chinese patients with CD from real-world experience. Moreover, we hypothesized that the functional composition of microbiota in the gut is of importance for ADA therapy outcomes in CD patients. We aimed to characterize fecal microbiota alteration trends during ADA therapy, identify the bacterial taxa those are associated with clinical and endoscopic response to ADA treatment, and investigate potential microbiome-based predictors for ADA efficacy at early stages.

## Materials and Methods

### Study population

Data were extracted from the databases of the Center for IBD research, Department of Gastroenterology, Shanghai Tenth People’s Hospital of Tongji University, and Department of Gastroenterology in Shanghai General Hospital, which are ongoing primary care databases of IBD patients who are both registered and in follow up in these centers.

For ADA treatment, data from 1 January 2020 to 1 January 2021 were included. The index period ended on 31 March 2021. The inclusion criteria were 1) age >18 years and 2) ileal, colonic, or ileocolonic CD diagnosed using clinical, laboratory, radiological, endoscopic, and histological evidence. The exclusion criteria were 1) age <18 years, 2) received ADA but not evaluated with endoscopic test in 12 weeks, and 3) pregnancy or lactation status. The enrolled participants completed specified physical, instrumental, and biochemical examinations before starting ADA therapy. ADA treatment decisions for individuals were proposed after discussion with our multidisciplinary treatment group. All recruited patients with CD received ADA of 160 mg at week 0, 80 mg at week 2, and 40 mg at weeks 4, 6, 8, 10, and 12 and were followed up for 12 weeks. The demographic and clinical characteristics of the participants were collected according to follow-up medical materials. The Montreal classification was used to categorize the disease location and behavior (Satsangi et al., 2006).

### Sample collection

As a prerequisite to patients’ approval, fecal samples were collected from CD patients who did not receive any antibiotics or probiotics within the 4 weeks prior to ADA treatment. Fecal samples were frozen at −80°C within 15 min after sampling until further DNA extraction and fecal calprotectin (FC) measurement at baseline and week 12. In addition, patients' blood samples were collected immediately before dosing at specified time points and analyzed by the enzyme-linked immunosorbent assay to determine anti-adalimumab antibody (AAA) levels, ADA serum concentrations, and serum TNF concentrations (WuXi AppTec, Shanghai, China).

### Clinical outcome evaluation

The Crohn’s disease activity index (CDAI) was used to measure clinical disease activity, and CDAI evaluation was applied to patients before ADA therapy at weeks 0 and 12 (Best et al., 1976). Clinical activity was defined as CDAI ≥150. Clinical remission (CR) was defined as CDAI <150. All patients underwent ileocolonoscopy or enteroscopy at baseline (week 0) and week 12. The Crohn’s Disease Endoscopic Index of Severity (CDEIS) scores were calculated by two experienced physicians to assess endoscopic activity (Vuitton et al., 2016). Responders in this study were defined as patients with a positive endoscopic response (ER), which was defined as CDEIS reduction ≥50% of the original score. Nonresponders were defined as patients with a decrease in CDEIS <50% of the original score or even worse. Mucosal healing (MH) was assigned a CDEIS score between 0 and 3. The primary outcome was to compare the efficacy of ADA in CD patients with different disease locations using the data from real-world experience, and the composition of fecal microbiota from selected patients with CD before and after receiving ADA therapy.

### Fecal calprotectin measurement

About 100 mg of feces was extracted and mixed with 5 ml fecal extraction buffer in a closed tube. After centrifugation, samples of the supernatant were tested for FC using the PhiCal Calprotectin ELISA Kit (Immundiagnostik AG, Bensheim, Germany) according to the manufacturer’s instructions.

### Immunogenicity and pharmacokinetics

Twenty-five patients' blood samples were collected immediately before ADA injection at certain time points and were analyzed by the enzyme-linked immunosorbent assay to determine anti-adalimumab antibody (AAA) levels (WuXi AppTec, Shanghai, China). A patient was considered AAA positive if they had ⩾1 AAA concentration >20 ng/ml. Samples with a quantifiable AAA concentration (>20 ng/ml) underwent confirmatory assays to determine the specificity of the AAA response. The ADA serum concentration was measured by using a validated electrochemiluminescence ligand–binding assay method. The lower limit of quantification for ADA was established at 50 ng/ml in undiluted serum.

### Bacterial DNA extraction and polymerase chain reaction amplification

Bacterial DNA extraction was performed from the fecal samples using the EZNA^®^ Soil DNA Kit (Omega Bio-tek, Norcross, GA, United States), according to the manufacturer’s instructions. The NanoDrop 2000 UV-vis spectrophotometer (Thermo Scientific, Wilmington, Delaware, United States) was used to determine the final DNA concentration and purity, and DNA quality was checked using 1% agarose gel electrophoresis. The V3-V4 hypervariable regions of the bacterial 16S rRNA gene were amplified with the primers 338F (5’-ACT​CCT​ACG​GGA​GGC​AGC​AG-3’) and 806R (5’-GGACTACHVGGGTWTCTAAT-3’) by a thermocycler PCR system (GeneAmp 9700, ABI, Foster, CA, United States). The PCR reactions were conducted using the following program: 3 min of denaturation at 95°C, 27 cycles of 30 s at 95°C, annealing at 55°C for 30 s, elongation at 72°C for 45 s, and a final extension at 72°C for 10 min. The PCR reactions were performed in triplicate; each 20 μL reaction mixture comprised 4 μL of 5× FastPfu Buffer, 2 μL of 2.5 mM deoxyribonucleotide triphosphate, 0.8 μL of each primer (5 μM), 0.4 μL of FastPfu Polymerase, and 10 ng of template DNA. The resultant PCR products were extracted from a 2% agarose gel, purified using the AxyPrep DNA Gel Extraction Kit (Axygen Biosciences, Union City, CA, United States), and quantified using Qubit 4 (Thermo Fisher, United States) according to the manufacturer’s protocol.

### 16S rRNA gene sequencing

Cloned libraries were pooled in equimolar amounts and sequenced on the Illumina MiSeq PE300 platform (Illumina Corporation, San Diego, CA, United States) at Shanghai Honsun Biotechnology Co., Ltd. (Shanghai, China). The raw sequencing reads were demultiplexed, quality filtered by fastp (version 0.21.0; https://github.com/OpenGene/fastp), and merged by FLASH (version 1.2.7; http://ccb.jhu.edu/software/FLASH/) with the following criteria: 1) the 300-bp reads were truncated at any site receiving an average quality score of <20 over a 50-bp sliding window, and the truncated reads shorter than 50 bp were discarded; 2) exact barcode matching, two nucleotide mismatch in primer matching, and reads containing ambiguous characters were removed; 3) only overlapping sequences longer than 10 bp were assembled according to their overlapped sequence. Reads that could not be assembled were discarded. USEARCH (version 7.1; http://www.drive5.com/usearch/) was used to identify operational taxonomic units (OTUs) based on 97% sequence identity and check the sequence quality. The representative sequences obtained for each OTU were compared with Silva 132 (http://www.arb-silva.de) to obtain taxonomic information with the RDP Classifier (version 2.2; http://sourceforge.net/projects/rdp-classifer/).

### Bioinformatics analysis

Rarefaction was performed and used to compare the relative abundance of OTUs across samples. Alpha diversity indices (observed OTUs, Chao, and Shannon) were determined with the mothur software (version 1.30.1; https://mothur.org/). The principal coordinates analysis (PCoA) and nonmetric multidimensional scaling (NMDS) analysis were performed to evaluate the beta diversity distance matrix using vegan and ggplot2 packages in R software (version 3.6.3; https://www.r-project.org/), and the adonis analysis was performed to test the reliability of PCoA and NMDS clusters using QIIME (version 1.9.1; http://qiime.org/index.html). The characterization of microorganismal features differentiating the gut microbiota was performed using the linear discriminant analysis (LDA) effect size (LEfSe) method (http://huttenhower.sph.harvard.edu/lefse/), which emphasizes both statistical significance and biological relevance. With a normalized relative abundance matrix, LEfSe uses the Kruskal–Wallis rank sum test to detect features with significantly different abundances between assigned taxa and performs LDA to estimate the effect size of each feature. Furthermore, a generalization of the (logarithmic) fold change was evaluated for each genus. This quantity was used to be applied to the genomic sequencing data such as RNA sequencing (RNA-seq) and Global run-on sequencing (GRO-seq) widely and now has been further improved for better resolution of sparse microbiome profiles (Feng et al., 2012). The generalized fold change was calculated as the average difference between predefined quantiles of the logarithmic abundance between ADA-response and ADA-nonresponse distributions. A significant alpha at 0.05 and an effect size threshold of 2 were used for all biomarkers discussed in this study. The functions of the gut microbiome were inferred from 16S rRNA sequences using PICRUSt 2.0 (Phylogenetic Investigation of Communities by Reconstruction of Unobserved States) (version 2.4.1; https://github.com/picrust/picrust2). With the predicted metagenome, the Kyoto Encyclopedia of Genes and Genomes (KEGG) pathway functions were categorized per level (from KEGG pathway level 1 to KEGG pathway level 3).

### Statistical analysis

All statistical analyses were performed by using the IBM Statistical Package for Social Sciences (SPSS) 20.0 software (IBM Corporation, Armonk, NY, United States) and GraphPad Prism version 7.0 (GraphPad Software, La Jolla, CA, United States). The quantitative data are shown as mean ± standard deviation (SD) or median [interquartile ratio (IQR)]. The *t*-test was used to compare the means of two samples in the independent samples. The resulting *p*-values were adjusted for multiple testing according to the Benjamini–Hochberg procedure. Pearson’s chi-squared test was performed for comparisons of different groups. When the chi-squared test was not appropriate (in case the total number of observations was less than 20 or the number of frequency cells was less than 5), Fisher’s exact test was used to calculate an exact *p*-value with a small number of expected frequencies by using the Bonferroni adjustment. Multiple testing was applied during the differential abundance analysis of bioinformatics and the adjusted *p*-value was used as the filtering parameter.

### Ethical considerations

This study was approved by the Ethics Agency of the Shanghai Tenth People’s Hospital of Tongji University. The URL of the online registry is http://www.chictr.org.cn/index.aspx, and the registry number is ChiCTR2000036115.

## Results

### Characteristics of the study population

From 1 January 2020 to 1 March 2021, 115 patients with an established diagnosis of CD were selected for this study from the Inflammatory Bowel Disease Center of the Shanghai Tenth People’s Hospital of Tongji University and the Department of Gastroenterology, Shanghai General Hospital of Shanghai Jiao Tong University. Their clinical characteristics are detailed in Table 1. All CD patients received induction therapy with ADA once every 2 weeks until 12 weeks. Adverse effects were reported during follow-up as shown in Supplementary Table S1. During 12 weeks of ADA therapy, no patient discontinued ADA treatment because of serious adverse effects.

**TABLE 1:** Baseline characteristics of the 115 patients with Crohn’s disease.

Characteristics	n, frequency (%) or median (IQR)
Male	80 (69.6)
Age (years)	32.87 (16–68)
Crohn’s disease phenotype at indusion (Montreal classification)^a^	
L1 (ileal disease)	22 (19.1)
L2 (colonic disease)	18 (15.7)
L3 (ileocolonic disease)	73 (63.5)
L4 (upper gastrointestinal disease)	2 (1.7)
B1 (inflammatory predominant)	91 (79.1)
B2 (stricturing disease)	24 (20.9)
B3 (penetrating disease)	0
P^b^	56 (48.7)
Previous intestinal resection	26 (22.6)
Previous treatment with inflizimab	31 (27.0)
Concomitant drugs at the time of inclusion 5-aminosalicylates	3 (2.6)
Thiopurines	7 (6.1)
Enteral nutrition^c^	8 (7.0)
No concomitant drugs	97 (84.3)
Clinical activity^d^	102 (88.7)

a Satsangi et al.

b Perianal disease modifier added to B when concomitant perianal disease is present.

c Total enteral nutrition included.

d Crohn’s disease activity index >150 and Crohn’s disease endoscopic index of severity >3.

### Adalimumab therapy induces clinical remission and mucosal healing in mild to moderate Crohn’s disease patients

**FIGURE 1:**
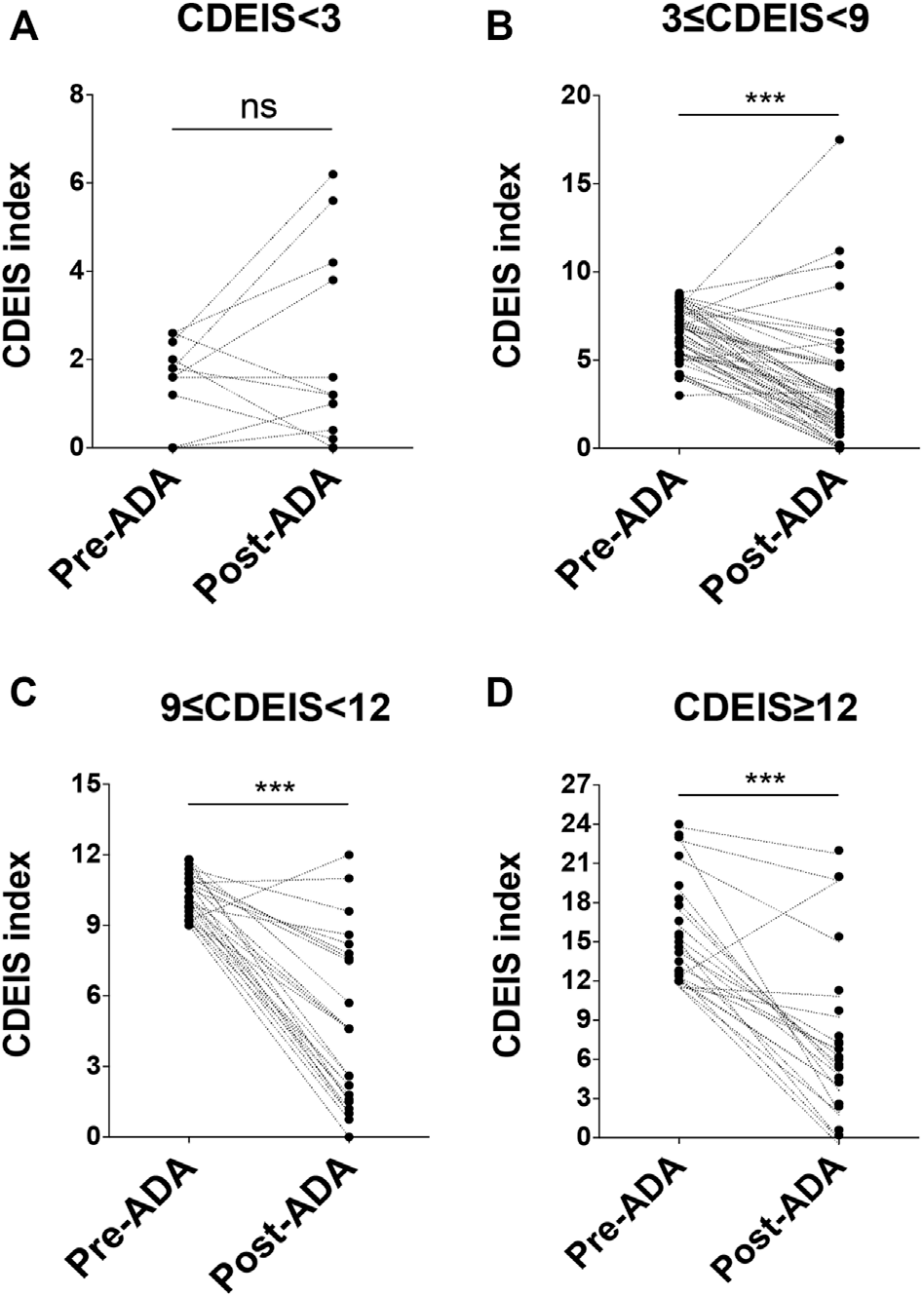
Details of patients' response to ADA at week 12 according to the CDEIS scores. At baseline, patients were divided into four groups. They were quiescent CD (CDEIS <3) **(A)**, mildly active CD (3 ≤ CDEIS < 9) **(B)**, moderately active CD (9 ≤ CDEIS < 12) **(C)**, and severely active CD (CDEIS ≥12) **(D)**. ADA, adalimumab; CDEIS, Crohn’s Disease Endoscopic Index of Severity; ns, not significant; ***p < 0.001.

Clinical, biochemical, and endoscopic examinations were recorded before and after ADA therapy. At week 12, CR was achieved in 70.6% (72/102) of the patients with active CD. As shown in Table 2, a total of 47.1% (48/102) of the patients with active CD attained MH (Figure 1A). When taking into consideration the severity of disease with the CDEIS score, we found that 56.6% (30/53) of the patients with mildly active CD (3 ≤ CDEIS < 9) and 48.0% (12/25) of the moderately active CD patients (9 ≤ CDEIS < 12) attained MH (Table 2). These differences were both statistically significant between pre- and post-ADA treatments (Figures 1B,C). Interestingly, though only 25.0% (6/24) achieved MH among severely active CD patients (CDEIS ≥12), the increase was still significant when compared with post-ADA to pre-ADA treatments (Figure 1D). Given that ADA efficacy varied with different active stages of CD patients, we further analyzed the remission rates between different groups and found that the difference was significant (*χ* ^2^ = 9.483, *p* = 0.024) (Table 2), indicating that patients with mild to moderate CD (3 ≤ CDEIS < 12) benefit the most from ADA treatment. In addition, 69.2% (9/13) of the quiescent CD patients (CDEIS <3) maintained MH. Meanwhile, we found no significant difference in quiescent CD patients between before and after ADA treatments, suggesting that ADA might be one of the therapeutic strategies recommended for quiescent CD patients to obtain sustained remission (Figure 1A). The values and variations of the CDAI, FC, BMI, CRP, and ESR from week 0 to week 12 (the end of follow-up) were also analyzed as shown in Table 2.

**TABLE 2:** Patient demographics of before and after adalimumab treatment.

	Before	After	*p* value	Pearson χ2	*p* value
CDEIS, n (%)^a^					
<3	13	9/13 (69.2)	0.432		
3–9	53	30/53 (56.6)	<0.001		
				9.483	0.024
9–12	25	12/25 (48.0)	<0.001		
≥2	24	6/24 (25.0)	<0.001		
CDAI, mean (SD)	230.3 ± 130.3	142.8 ± 80.2	<0.001		
FC, mean (SD), ug/g	637.8 ± 56.1	415.8 ± 73.2	0.016		
BMI, mean (SD), kg/m^2^	19.9 ± 3.7	20.8 ± 4.0	0.137		
CRP, mean (SD), mg/L	22.8 ± 31.8	15.0 ± 20.7	0.028		
ESR, mean (SD), mm/h	36.2 ± 23.6	21.0 ± 14.6	<0.001		

a n(%) means the numbers and the frequency of CD patients that achieved mucosal healing in each group according to CDEIS.

### Adalimumab efficacy is not associated with disease location and previous medicine in Crohn’s disease

With regard to disease location, CD patients with L1 (ileal disease) achieved MH by 45.5% (10/22), while patients with L2 (colonic disease) and L3 (ileocolonic disease) attained MH by 55.6% (10/18) and 49.3% (37/75), respectively. No significant difference was found among remission rates in CD patients with different disease locations (*χ* ^2^ = 0.409, *p* = 0.815) (Table 3). Thereby, it is suggested that there seems no relationship between the efficacy of ADA and disease locations of CD. In order to explore whether the efficacy of ADA is influenced by previous drugs, we performed a statistical analysis of the remission rates in CD patients with or without previous treatments. As shown in Table 3, 56.1% (37/66) of the CD patients with no previous treatment attained MH. While 35.5% (11/31) of the patients who failed IFX treatment were still responsive to ADA, and patients who failed steroids treatment were responsive to ADA by 27.8% (5/23). Interestingly, although the remission rate seemed to be the highest in patients with no previous treatment, there were no statistically significant differences among remission rates in patients with or without previous therapies (Fisher’s exact test = 8.133, *p* > 0.0125) (Table 3). It has been indicated that ADA could be a good choice to be applied to CD patients who have failed IFX or steroid treatment, besides patients without previous treatment.

**TABLE 3:** Percentage of patients response to adalimumab at week 12.

Variable	Total number	Respond n, frequency (%)		
Disease location, n(%)^a^			Pearson χ^2^	*p* value
L1 (ileal disease)	22	10 (45.5)	0.409	0.815
L2 (colonic disease)	18	10 (55.6)		
L3 (ileocolonic disease)	75	37 (49.3)		
L4 (upper gastrointestinal disease)	2	0		
Previous failed treatment			Fisher’s exact test	*p* value
No treatment	66	36 (54.5)	8.133	0.041^b^
Infliximab	26	8 (30.8)		
Steroids	12	4 (33.3)		
Infliximab & Steroids	11	2 (18.2)		
CD surgical history				
Any surgery before baseline	26	15 (57.7)		
Surgery within 12 w of baseline	8	NA		

a Patients could have multiple CD locations. Patients with both colonic and ileal CD were categorized as ileal-colonic. The locations of colonic, ileal, and ileal-colonic did not overlap. The locations of ileal-colonic ovedaped with upper gastrointestinal disease.

b No statistical significance of paired comparison due to p > a (0.0125) after Bonferroni adjustment.

### Adalimumab serum concentration as a biomarker does not predict adalimumab response

**FIGURE 2:**
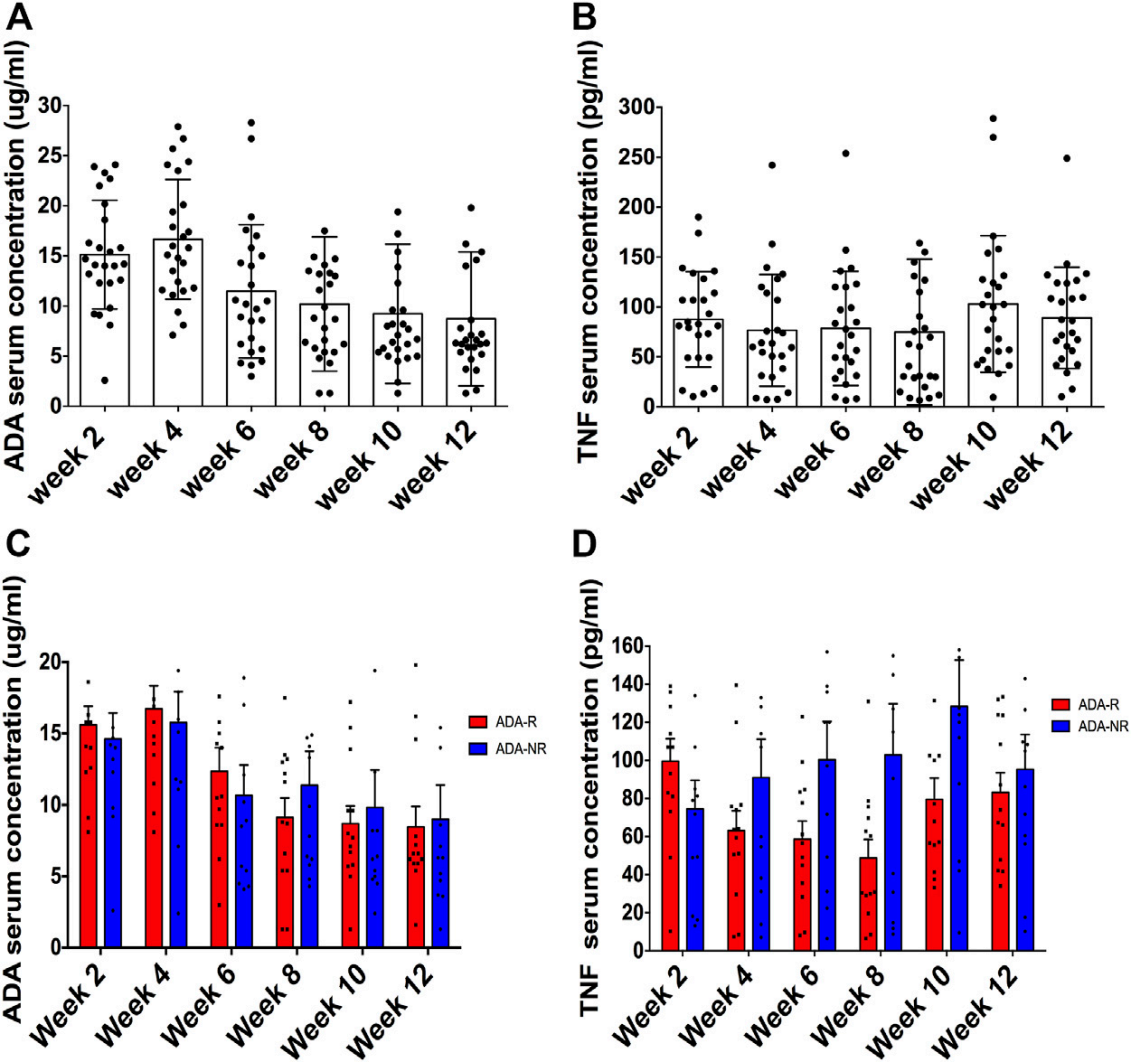
The ADA serum concentration is not a good biomarker for predicting ADA response. Of 25 patients, ADA serum concentrations were all over the lowest effective blood drug concentration (5 ug/ml) **(A)**. TNF serum concentrations presented no obvious changes along ADA treatment **(B)**. Furthermore, no significant differences between ADA-R and ADA-NR ADA were observed among serum concentrations **(C)** and serum concentrations of soluble TNF **(D)**. ADA-R, adalimumab responsive group; ADA-NR, adalimumab nonresponsive group.

As a prerequisite to patients’ approval, blood samples of 25 CD patients were collected pre-ADA injection and before dosing immediately, every 2 weeks until week 12. As shown in Table 4, ADA serum concentrations were all over the lowest effective blood drug concentration (5 ug/ml), which are shown in Figure 2A in detail. Of the 25 patients, most of them were AAA negative throughout the 12 weeks, and only 1 patient presented as AAA positive at week 8 and another at week 12, thus 2 patients in total reported being AAA positive in 12 weeks (Table 4). As regards TNF serum concentrations, we observed that there were no obvious changes along ADA treatment (Table 4 and Figure 2B). Since our previous study demonstrated that anti-TNFs binding transmembrane TNF (tmTNF) is strongly involved in treatment response *via* reverse signaling (Fang et al., 2018), serum concentrations of soluble TNF may not be considerably a predictive index according to our data here. Interestingly, we also found that there were no significant differences between ADA-R and ADA-NR among ADA serum concentrations and serum concentrations of soluble TNF, indicating that there may be more characteristic predictive indexes other than those for ADA response evaluation (Figures 2C,D).

**TABLE 4:** ADA relatated indicators monitoring in patients with CD, *n* = 25.

	Week 2	Week 4	Week 6	Week 8	Week 10	Week 12
ADA serum concentration, mean (SD), ug/ml	15.1 ± 5.4	16.7 ± 6.0	11.5 ± 6.6	10.2 ± 6.7	9.2 ± 7.0	8.7 ± 6.7
AAA positive, n	0	0	0	1	1	2
TNF serum concentration, mean (SD), pg/ml	87.6 ± 48.0	76.5 ± 56.1	78.7 ± 57.2	74.7 ± 73.1	103.0 ± 68.3	89.1 ± 50.6

ADA, adalimumab; AM, anti-adalimumab antibody; TNF, tumor necrosis factor.

### Gut microbiota alteration trends during adalimumab therapy

**FIGURE 3:**
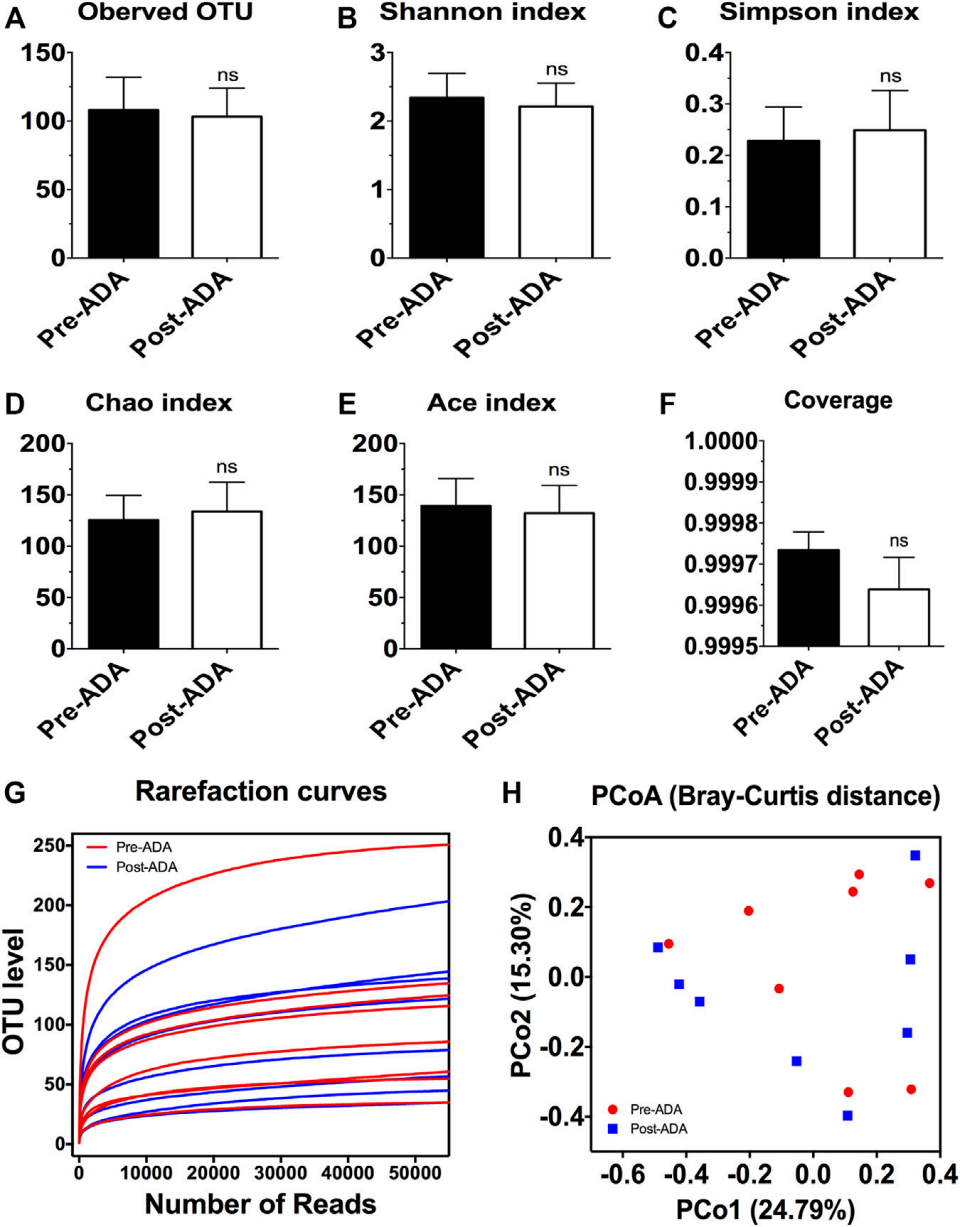
The alpha and beta diversity analysis of gut microbiota in CD patients during ADA therapy. Alpha diversity indices as estimated using the number of observed OTU **(A)**, Shannon index **(B)**, Simpson index **(C)**, Chao index **(D)**, ACE index **(E)**, coverage **(F)**, and rarefaction curves **(G)**; beta diversity of gut microbiota calculated *via* PCoA across all pre- and post-ADA samples using Bray–Curtis distance **(H)**; ns, not significant.

**FIGURE 4:**
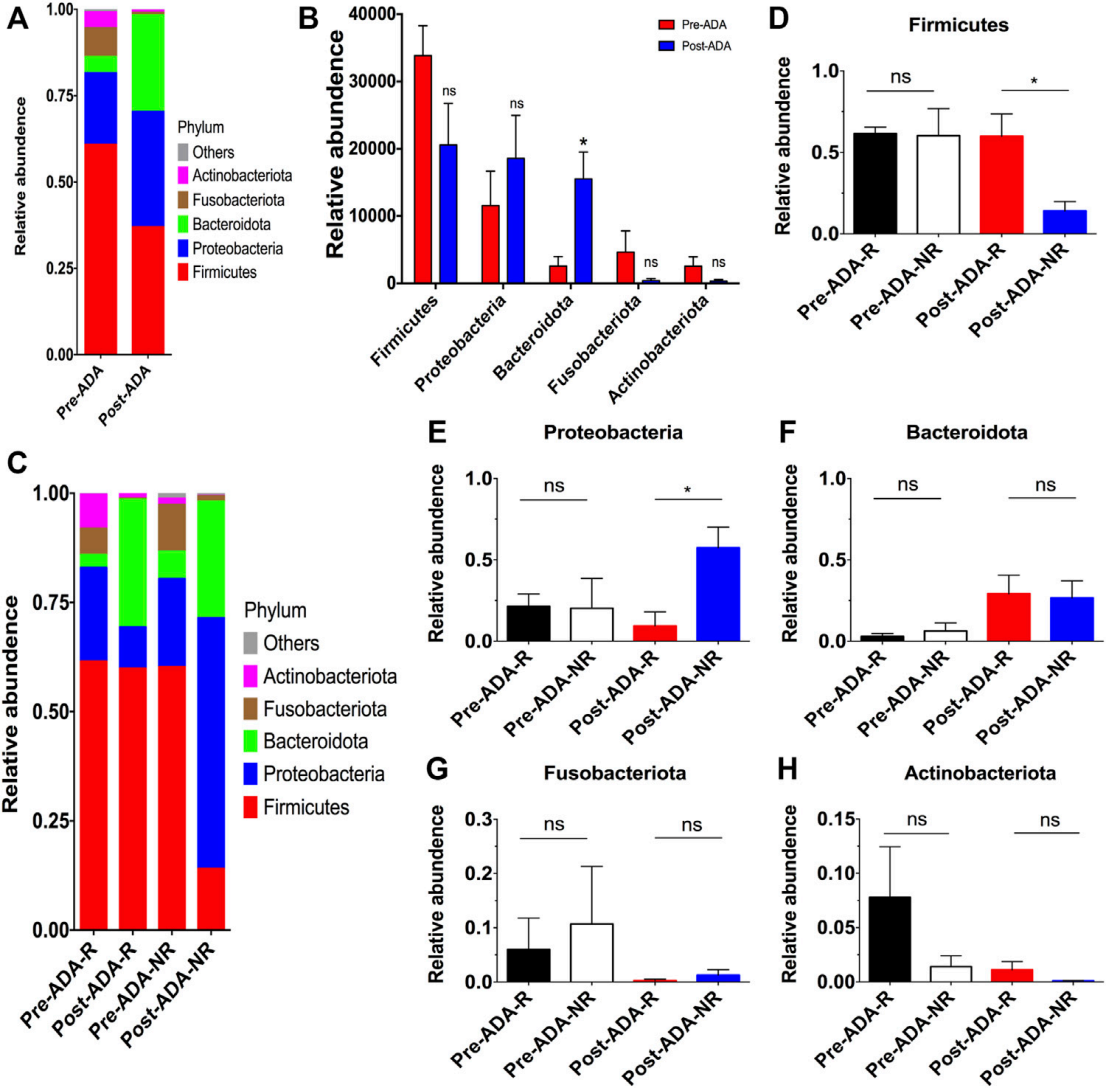
Taxonomic differences in the fecal microbiota of patients with CD between different subgroups during ADA therapy. Relative proportions of bacterial phyla were compared between pre-ADA and post-ADA **(A)**, and statistically analyzed among typical phyla **(B)**. Meanwhile, the relative abundance of bacterial phyla was also compared in between Pre-ADA-R, Post-ADA-R, Pre-ADA-NR, and Post-ADA-NR **(C)**. **(D)** Firmicutes, **(E)** Proteobacteria, **(F)** Bacteroidetes, **(G)** Fusobacteriota, and **(H)** Actinobacteriota; *p < 0.05; ns, not significant. Pre-ADA-R, responsive pre-adalimumab samples; Post-ADA-R, responsive post-adalimumab samples Pre-ADA-NR, nonresponsive pre-adalimumab samples; Post-ADA-NR, nonresponsive post-adalimumab samples.

Recent evidence have suggested that certain gut microbiota composition and function are specially associated with the clinical and endoscopic responses to IFX, providing potentially predictive biomarkers for IFX treatment decision-making (Zhuang et al., 2020). On account of this, we considered that there may be a window of opportunity allowing microbial biomarkers to predict and monitor ADA treatment outcomes. Therefore, a total of 889,888 high-quality 16S rRNA gene sequences were obtained from 16 fecal samples of 8 ADA-treated CD patients after quality trimming and chimera checking. Nutritional assessments such as the Nutritional Risk Screening-2002 (NRS-2002) and Patient-Generated Subjective Global Assessment (PG-SGA) were performed on these patients in order to exclude bias caused by nutritional differences. The analysis of alpha diversity differentiates neither the richness nor the diversity of pre- and post-ADA samples (Figures 3A–E). In addition, the Good’s coverage values were all more than 99.9%, and the rarefaction curves were gradually smooth for all sequences at different time points, indicating that the sequencing data were sufficient for investigating fecal microbiota (Figures 3F,G). However, the principal coordinates analysis (PCoA) calculated on Bray–Curtis dissimilarity did not differentiate the fecal microbial communities of pre- and post-ADA samples (Figure 3H). The RDP classifier was used to assess the taxonomy of fecal microbiota *via* taxon-dependent analysis. We performed taxonomic profile alterations to find potential ADA-associated biomarkers for predicting the treatment response. With regard to the abundant phylum level, the relative abundances of *Firmicutes*, *Fusobacteriota*, and *Actinobacteriota* were inclined to be reduced in patients with CD during ADA therapy, whereas a tendency of higher relative abundances of *Proteobacteria* and *Bacteroidota* were observed in post-ADA samples than pre-ADA ones (Figure 4A). We statistically analyzed the differences in relative abundances at the phylum level and found significantly higher relative abundances of *Bacteroidota* in post-ADA samples than in pre-ADA ones (Figure 4B).

Given that there were still a considerable number of patients who experienced unexpected therapeutic responses to ADA, we further compared the fecal microbiota in patients responsive and nonresponsive to ADA. Consequently, we observed higher relative abundances of *Firmicutes*, while reduced relative abundances of *Proteobacteria* in responsive post-ADA samples (Post-ADA-R) compared with the nonresponsive counterparts (Post-ADA-NR) (*p* < 0.05) (Figures 4C–E). Meanwhile, although the statistical analysis has shown no significant differences, we observed that there was a tendency of higher relative abundances of *Bacteroidota* and *Actinobacteriota* in Post-ADA-R than in Post-ADA-NR (Figures 4F,H) and that relative abundances of *Proteobacteria* tended to increase in Post-ADA-NR when compared with Post-ADA-R (Figure 4G).

### Gut microbiota alteration for adalimumab response

**FIGURE 5:**
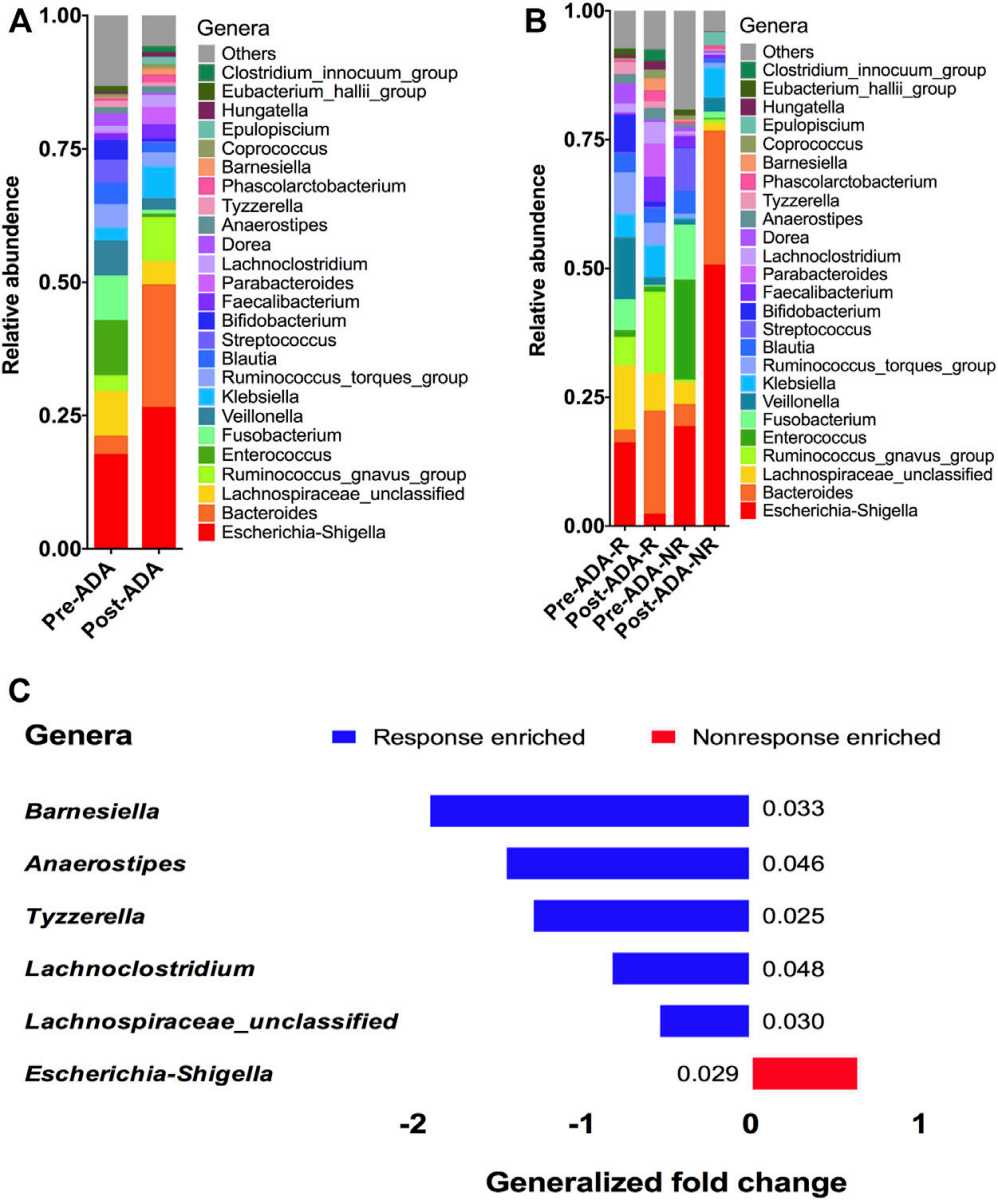
Relative proportions of bacterial genera were compared between pre-ADA and post-ADA **(A)** and were also compared in between Pre-ADA-R, Post-ADA-R, Pre-ADA-NR, and Post-ADA-NR **(B)**. The genera columns display the taxonomy information at the genus level. *p* values were computed using a two-sided blocked Wilcoxon rank-sum test and the exact *p* values are presented beside the barplots. Generalized fold change (see the Materials and Methods section) is indicated by color gradients. The generalized fold change >0, enriched in nonresponsive adalimumab samples; generalized fold change <0: enriched in responsive adalimumab samples. **(C)**. Pre-ADA-R, responsive pre-adalimumab samples; Post-ADA-R, responsive post-adalimumab samples; Pre-ADA-NR, nonresponsive pre-adalimumab samples; Post-ADA-NR, nonresponsive post-adalimumab samples.

At the genera level, 25 genera were identified with distinguishable differential abundances between gut communities of pre-ADA and post-ADA samples (Figure 5A). We further analyzed the relative abundances of these genera in comparison to responsive ADA samples (Pre-ADA-R, Post-ADA-R) and nonresponsive counterparts (Pre-ADA-R, Post-ADA-NR) (Figure 5B). Specifically, there were six genera with significant differential abundances in comparison to gut communities between responsive and nonresponsive counterparts. They were assigned as *Barnesiella*, *Anaerostipes*, *Tyzzerella*, *Lachnoclostridium*, *Lachnospiraceae_unclassified*, and *Escherichia–Shigella*. As shown in Figure 5C, the abundance of the five genera was increased in the responsive group when compared with its nonresponsive counterpart, which were assigned as *Barnesiella*, *Anaerostipes*, *Tyzzerella*, *Lachnoclostridium*, and *Lachnospiraceae_unclassified*. Meanwhile, the higher abundance of pathogenic bacteria (*Escherichia–Shigella*) was observed in the nonresponsive group than its responsive counterpart. All of these different genera may be potentially predictive biomarkers of ADA efficacy.

### Microbial functional changes during adalimumab therapy

**FIGURE 6:**
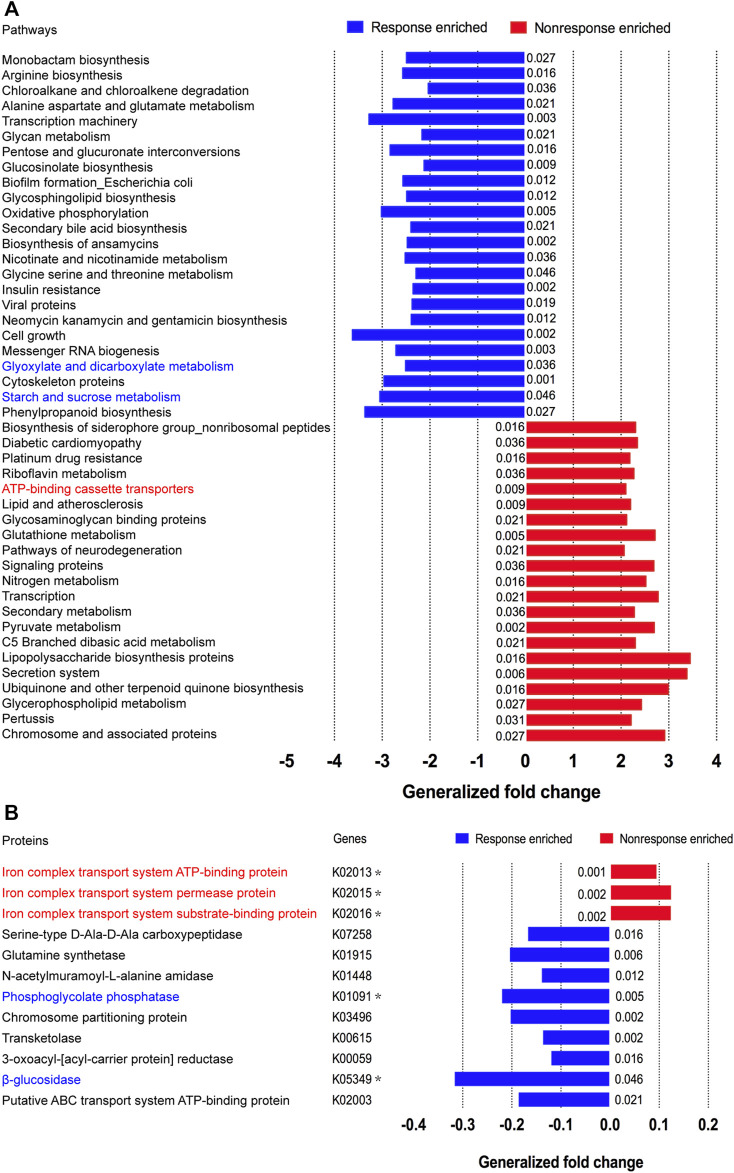
Microbial functional alterations in ADA response. The relative abundances of functional pathways **(A)** and bacterial genes **(B)** were compared between the nonresponsive adalimumab and responsive adalimumab samples. Differentially abundant pathways are highlighted in blue and red; p values were computed using a two-sided blocked Wilcoxon rank-sum test and the exact p values <0.05 are presented beside the barplots. Generalized fold change (see the Materials and Methods section) is indicated by color gradients. The \generalized fold change >0: enriched in the former; generalized fold change <0: enriched in the latter.

**FIGURE 7:**
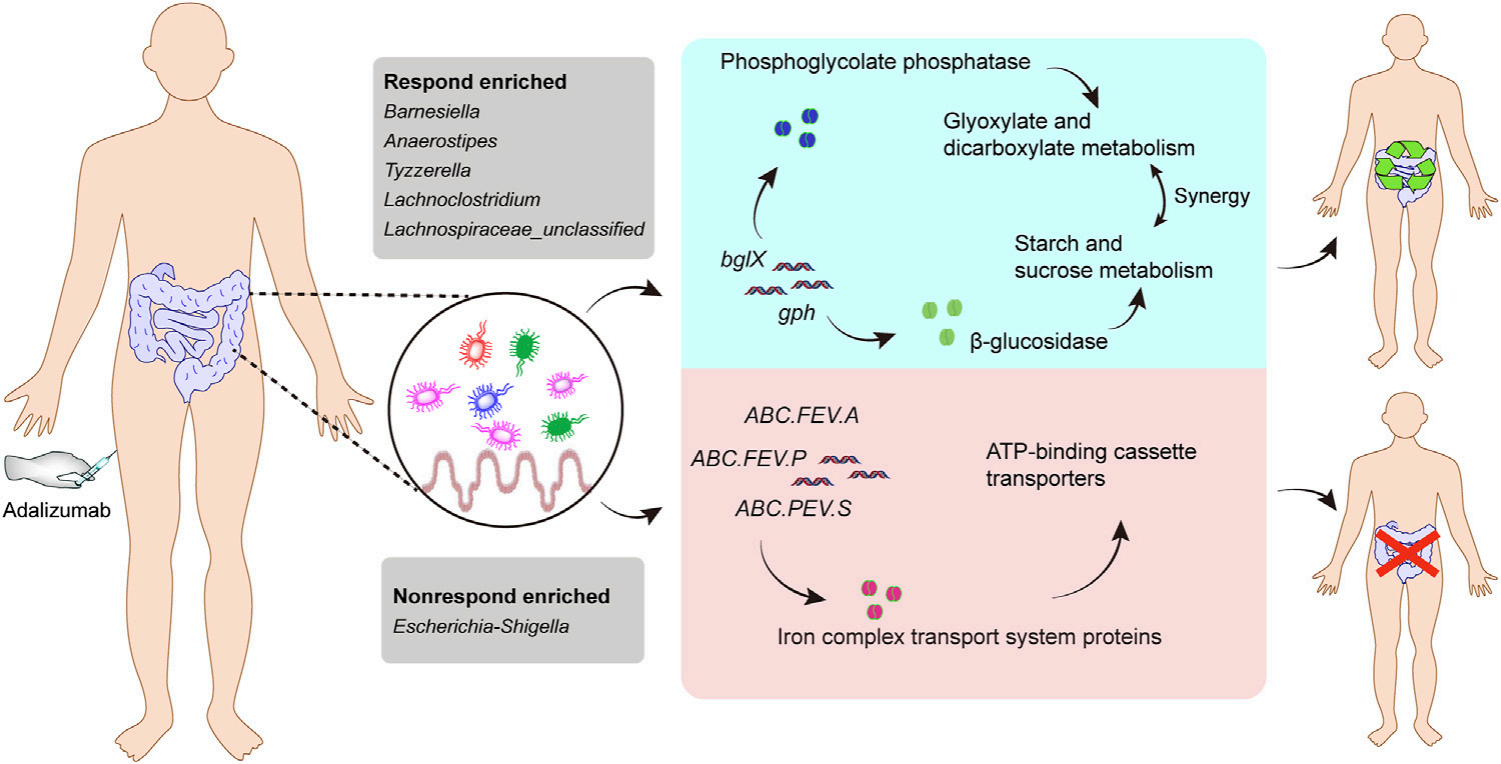
Gut microbiome–associated mechanism of ADA therapy response. The abundance of the five genera (*Barnesiella*, *Anaerostipes*, *Tyzzerella*, *Lachnoclostridium*, and *Lachnospiraceae*_*unclassified*) were increased in the responsive group when compared with their nonresponsive counterparts, while a higher abundance of pathogenic bacteria (*Escherichia–Shigella*) was observed in the nonresponsive group than in its responsive counterpart. The gene *bglX* encoding β-glucosidase and *gph* encoding phosphoglycolate phosphatase are both activated during ADA treatment, facilitating starch and sucrose metabolism and glyoxylate and dicarboxylate metabolism, respectively, thus contributing to ADA efficacy synergistically. Conversely, the enrichment of ABC.FEV.A, ABC.FEV.P, and ABC.FEV.S, which encode iron complex transport system proteins (i.e. ATP-binding protein, permease protein, and substrate-binding protein, respectively), alleviates ADA response in patients with CD *via* the pathway of ABC transporters.

**FIGURE 8:**
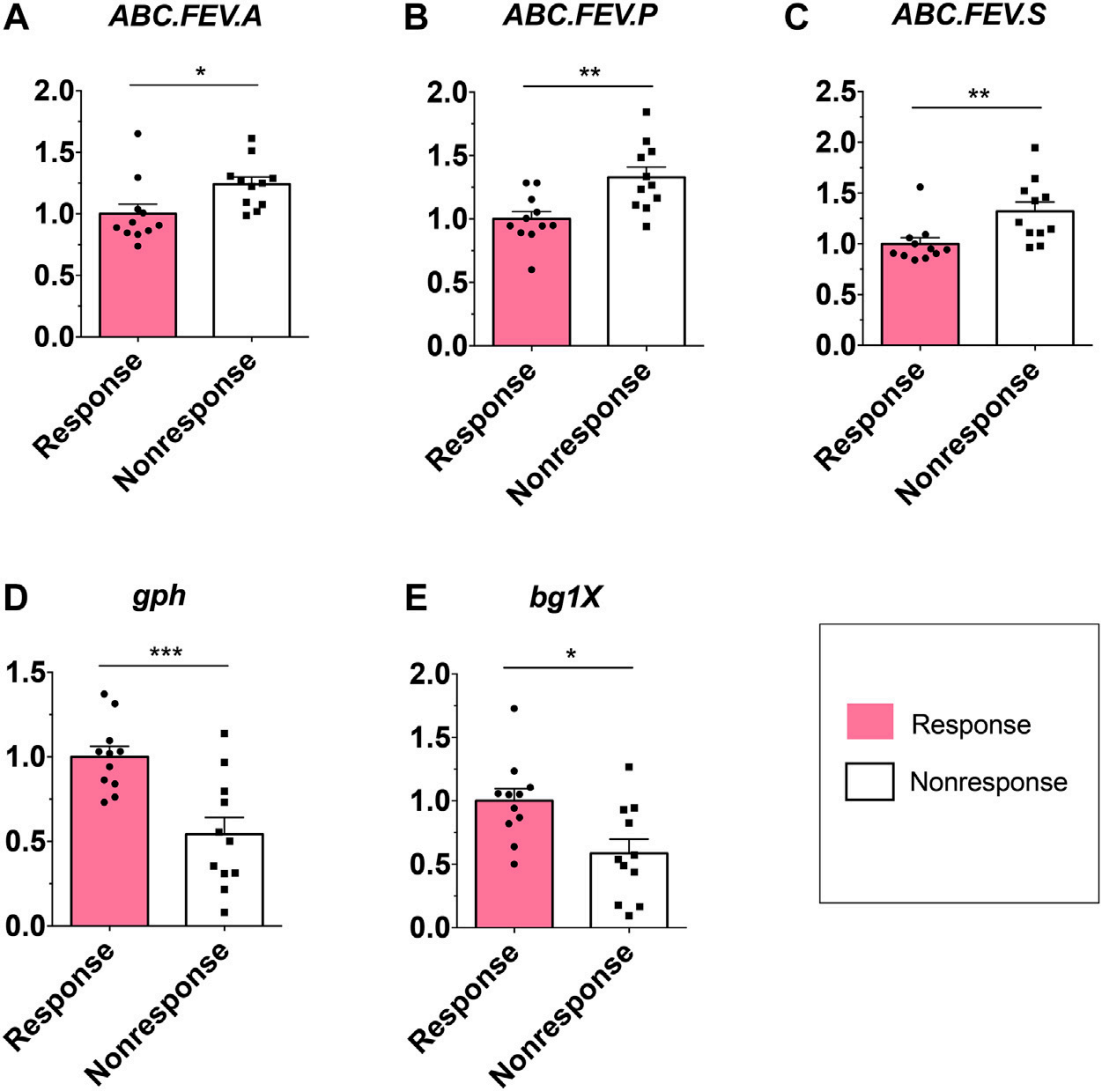
Relative abundance of candidate genes. Plotted values are qRT-PCR quantifications of bacterial genes in starch and sucrose metabolism, glyoxylate and dicarboxylate metabolism, and the ABC transporters system. The abundance of ABC.FEV.A, ABC.FEV.P, and ABC.FEV.S, and gph and bglX were compared between the ADA-responsive group (*n* = 11) and ADA-nonresponsive group (*n* = 11). *p < 0.05; **p < 0.01; ***p < 0.001.

Considering that the metabolic pathway contributed from fecal microbiota may be associated with ADA efficacy, we examined the gut microbiome–based functional alterations from taxonomic profiles referred to post-ADA administration by PICRUSt 2.0 and categorized at the level of the KEGG pathways. Totally, there were 45 differential pathways between the responsive group and its nonresponsive counterpart, which were clustered based on their generalized fold change scores (Figure 6A). Among these pathways, it is worth noting that the pathway of starch and sucrose metabolism which is dominated by β-glucosidase, a thermostable enzyme that is encoded by *bglX* (K05349), was enriched significantly in the ADA-responsive group when compared with the ADA-nonresponsive group*.* Consistently, the gene of *bglX* was activated notably in the ADA-responsive group as shown in Figure 6B. This indicated that *bglX* coding β-glucosidase contributes to the pathway of starch and sucrose metabolism, participating in the mechanism of ADA response in CD patients. Additionally, the gene of *gph* (K01091) was enriched in the ADA-responsive group as compared with the ADA-nonresponsive group (Figure 6B). Consistently, the pathway of glyoxylate and dicarboxylate metabolism, which is dominated by *gph* encoding phosphoglycolate phosphatase, was enriched in the responsive group as shown in Figure 6A. Notably, the abundance of ATP-binding cassette (ABC) transporters was significantly enriched in the ADA-nonresponsive group when compared with the -responsive group (Figure 6A), which indicated that the pathway of ABC transporters participates in reducing ADA efficacy. As major contributors to ABC transporters, there are three enzymes assigned as iron complex transport system ATP-binding protein, iron complex transport system permease protein, and iron complex transport system substrate-binding protein, which are encoded by the genes *ABC.FEV.A* (K02013), *ABC.FEV.P* (K02015), and *ABC.FEV.S* (K02016), respectively. These three genes were consistently enriched in the ADA-nonresponsive group when compared with the ADA-responsive group (Figure 6B). Taken together, it is suggested that *bglX* encoding β-glucosidase and *gph* encoding phosphoglycolate phosphatase are both activated during ADA treatment, facilitating starch and sucrose metabolism and glyoxylate and dicarboxylate metabolism, respectively, thus contributing to ADA efficacy synergistically. Conversely, the enrichment of *ABC.FEV.A*, *ABC.FEV.P*, and *ABC.FEV.S*, which encode iron complex transport system proteins (i.e., ATP-binding protein, permease protein, and substrate-binding protein) reduces ADA efficacy in patients with CD *via* the pathway of ABC transporters (Figure 7). A previous study proposed that the enterotypes, driven by species composition, indicate the existence of a limited number of host–microbial symbiotic states that might respond differently to drug administration (Arumugam et al., 2011). However, we identified three clusters as enterotypes and found no relationship between enterotypes and the efficacy of ADA (Supplementary Figure S1). Furthermore, we collected new samples and validated the abundance of the aforementioned key genes using qRT-PCR. The genes of *ABC.FEV.A*, *ABC.FEV.P*, and *ABC.FEV.S* were all enriched in the ADA-nonresponsive group, while the genes *gph* and *bglX* were enriched in the ADA-responsive group, which are consistent with the PICRUSt 2.0 results (Figures 8A–E).

Collectively, the data in this study demonstrate that ADA could markedly induce MH in patients with mildly to moderately active CD, besides maintaining sustained MH in quiescent CD patients from real-world experience. Furthermore, we assessed fecal microbiota of active CD patients who were responsive or nonresponsive to ADA and preliminarily analyzed gut microbiome–based functional alterations during ADA treatment. We found that elevation of *bglX* encoding β-glucosidase and *gph* encoding phosphoglycolate phosphatase by gut microbiota may serve as contributors in facilitating ADA efficacy, and conversely, the enrichment of *ABC.FEV.A*, *ABC.FEV.P*, and *ABC.FEV.S* encoding iron complex transport system proteins in the ABC transporters pathway may potentially reflect treatment failure in ADA-nonresponsive individuals. Our study provides credible treatment recommendations for ADA through real-world experience–based data and presents potential gut microbiome–associated biomarkers for predicting ADA response.

## Discussion

This study collected data from real-world experience of ADA therapy in Chinese patients with CD and demonstrated that ADA is effective in sustaining quiescent (CDEIS <3) and mildly to moderately active (3 ≤ CDEIS <12) states in CD patients, and the efficacy of ADA is not associated with disease location and previous medicine in CD. Moreover, the data in this study show that serum concentrations of soluble TNF and the ADA serum concentration may not be considerably predictive indexes for ADA response evaluation. Instead, certain gut microbiota may serve as a potential biomarker to predict ADA efficacy and gut microbiome–based functional alterations may contribute to facilitate ADA response and also compensate for feedback inhibition in ADA-nonresponsive individuals.

As a humanized monoclonal antibody, ADA has been shown to induce and maintain remission in global clinical trials of patients with CD (Hanauer et al., 2006; Jilani and Akobeng, 2007). However, it was not applied to Chinese CD patients until 1 January 2020. Though there was a randomized, multicenter, phase III study in China before that showed adalimumab markedly induced clinical remission in Chinese patients with moderately to severely active CD (Chen et al., 2020), there is still a lack of data on real-world experience about the efficacy and safety of ADA. Our study focused on MH according to endoscopic features and found considerable number of patients with active CD, especially mildly to moderately active CD patients, attained MH, which was not related with disease location or previous drug usage. Meanwhile, our study also observes that ADA maintained sustained remission in quiescent CD patients significantly. Given that rare cases of AEs have been reported in clinical practice, ADA may be reasonably believed to be a good choice for not only mildly to moderately active CD patients but also quiescent CD patients. However, it is not recommended for severely active CD patients (CDEIS ≥12) according to our data, which may be due to its administration route and in achieving effective concentration slowly to a certain extent. It has been largely accepted that administration timing is among the best clinical outcome predictor for adalimumab administration in Crohn’s disease (Feuerstein et al., 2021). Whereas we observed that the efficacy of ADA is not associated with previous medicine in CD, indicating that ADA could be recommended for CD patients who failed to respond to IFX or steroids over no treatment for the induction of remission besides maintenance of remission.

As an anti-TNF agent, IFX-predominant unwanted outcomes, such as losing response or primarily nonresponse, can be mostly explained by inadequate drug concentrations or the development of excessive antibodies to IFX. Profiting from the characteristic of a humanized monoclonal antibody and stable serum concentration, ADA may serve as a second anti-TNF therapy for pharmacokinetic failure in IFX treatment, besides primary treatment, in CD patients. Though no excessive antibodies to ADA have been reported in this study, there are still up to nearly 30% of CD patients who show no clinical benefit following ADA induction. Our observation reassuringly suggests that a high concentration of serum soluble TNF level and a low titer of ADA serum concentration are not associated with ADA efficacy. A previous study has reported that anti-TNFs offered a vital approach to the treatment of active CD patients through binding soluble TNF and tmTNF specifically with high affinity, and our previous study has demonstrated that anti-TNFs binding transmembrane TNF (tmTNF) is strongly involved in the treatment response *via* reverse signaling (Fang et al., 2018). Thus, tmTNF may be a predictive index for ADA efficacy more considerably than serum concentrations of soluble TNF, which needs an appropriate detection method for further study.

Given that it has been reported that specific alterations in gut microbiota composition and function could be used as potentially microbial biomarkers for the treatment response prediction in IBD (Kostic et al., 2014), we performed taxonomic profile alterations to find potential ADA-associated biomarkers for predicting the treatment response. We found a higher relative abundance of Wang et al. (2018); Zhuang et al. (2020). A comparison of fecal samples from the ADA-responsive and ADA-nonresponsive groups shows that the gut microbiota structure of the responsive group is clearly modified during the treatment period. In detail, protective bacterial genera such as *Barnesiella*, *Anaerostipes*, *Tyzzerella*, *Lachnoclostridium*, and *Lachnospiraceae* were found increased in the ADA-responsive samples*,* while pathogenic bacterial taxa such as *Escherichia–Shigella* genera were diminished contrarily (Zhuang et al., 2020; Alatawi et al., 2021; Mo et al., 2021; Chen et al., 2022). Such modifications have been reported to reverse the disturbance of the microecosystem, produce more short-chain fatty acids, and correct immunity imbalance (Cho et al., 2012; Meehan and Beiko, 2014), indicating that these bacteria may serve as potential microbiota biomarkers for predicting ADA treatment responses.

We found that elevation of *bglX* encoding β-glucosidase and *gph* encoding phosphoglycolate phosphatase by gut microbiota may serve as contributors in facilitating ADA efficacy, and conversely, enrichment of *ABC.FEV.A*, *ABC.FEV.P*, and *ABC.FEV.S* encoding for iron complex transport system proteins in the ABC transporters pathway may potentially reflect treatment failure in ADA-nonresponsive individuals. Bacteria rely on ABC transporters for the import of various nutrients. In vertebrates, accessibility of iron is restricted for bacterial pathogens residing inside the host, as it is attached to either heme molecules or other circulatory proteins. Therefore, there is always an ongoing battle between the host system and pathogens, known as “nutritional immunity” (Huang and Wilks, 2017). To capture the bound iron from the human hosts, many bacteria secrete siderophores to scavenge Fe^3+^ by forming soluble ferric–siderophore complexes, which can then be actively taken up *via* various ABC transporters (Kramer et al., 2020). Iron homeostasis is of importance for both hosts and their associated microbiota, which has been highlighted by anemia caused by iron deficiency (Seyoum et al., 2021), local restriction of iron availability as part of the innate immune response (Scott et al., 2021), and stress adaptation (Xu et al., 2021). Considering this importance, iron complex transport system proteins in the ABC transporters pathway enriched in ADA-nonresponsive individuals may suggest a kind of iron pillage by bacteria during energy metabolism of the host, resulting in the loss of ADA efficacy. Moreover, various other gene activation and metabolic capacity modifications of the KEGG pathways were observed in the fecal microbiota of patients with CD during ADA therapy, suggesting certain microbial functional alterations may be closely associated with ADA treatment efficacy, but independent exploration is required.

Taken together, we identified promising microbial-derived markers for distinguishing ADA-response from ADA-nonresponse, which may contribute to the noninvasive evaluation of response to ADA treatment. Furthermore, we proposed that the *bglX* coding β-glucosidase–mediated starch and sucrose metabolism and glyoxylate and dicarboxylate metabolism mediated by *gph* encoding phosphoglycolate phosphatase facilitated ADA response synergistically. Conversely, *ABC.FEV.A*, *ABC.FEV.P*, and *ABC.FEV.S* encoding for iron complex transport system proteins in the ABC transporters pathway may potentially reflect treatment failure in ADA-nonresponsive individuals.

Limitations: This study was a two-center cohort study of patients with active CD, and a relatively small sample size was involved in the study. This trial did not evaluate the maintenance of response or long-term immunogenicity of ADA. The lack of a validation data set for further mechanistic investigation exists in this study.

## Summary

This study focused on the data from real-world experience and demonstrated that adalimumab (ADA) could markedly induce mucosal healing (MH) in Chinese patients with mildly to moderately active CD, besides maintaining sustained MH in quiescent CD patients. Furthermore, the study analyzed ADA response–associated gut microbiota and found that *bglX* encoding β-glucosidase and *gph* encoding phosphoglycolate phosphatase by gut microbiota may serve as contributors in facilitating ADA efficacy. Conversely, enrichment of *ABC.FEV.A*, *ABC.FEV.P*, and *ABC.FEV.S*, encoding for the iron complex transport system proteins in the ABC transporters pathway, may potentially reflect treatment failure in ADA-nonresponsive individuals. This study provides credible treatment recommendations for ADA through real-world experience–based data and presents potential gut microbiome–associated biomarkers for predicting ADA response.

## Data Availability

The datasets presented in this study can be found in online repositories. The names of the repository/repositories and accession number(s) can be found below: https://www.ncbi.nlm.nih.gov; PRJNA806451.

## References

[B1] Alatawi H. Mosli M. Saadah O. Annese V. Al-Hindi R. Alatawy M. (2021). Attributes of intestinal microbiota composition and their correlation with clinical primary nonresponse to anti-TNF-α agents in inflammatory bowel disease patients. Bosn. J. Basic Med. Sci. 22, 412–426. 10.17305/bjbms.2021.6436 10.17305/bjbms.2021.6436 | Google Scholar PMC9162754 34761733

[B2] Arumugam M. Raes J. Pelletier E. Le Paslier D. Yamada T. Mende D. R. (2011). Enterotypes of the human gut microbiome. Nature 473, 174–180. 10.1038/nature09944 PubMed Abstract | 10.1038/nature09944 | Google Scholar 21508958 PMC3728647

[B3] Best W. R. Becktel J. M. Singleton J. W. Kern F. Jr. (1976). Development of a Crohn's disease activity index. Gastroenterology 70, 439–444. 10.1016/s0016-5085(76)80163-1 PubMed Abstract | 10.1016/s0016-5085(76)80163-1 | Google Scholar 1248701

[B4] Chen B. Gao X. Zhong J. Ren J. Zhu X. Liu Z. (2020). Efficacy and safety of adalimumab in Chinese patients with moderately to severely active Crohn's disease: Results from a randomized trial. Ther. Adv. Gastroenterol. 13, 1756284820938960. 10.1177/1756284820938960 10.1177/1756284820938960 | Google Scholar PMC7370564 32733600

[B5] Chen L. Sun M. Wu W. Yang W. Huang X. Xiao Y. (2019). Microbiota metabolite butyrate differentially regulates Th1 and Th17 cells' differentiation and function in induction of colitis. Inflamm. Bowel Dis. 25, 1450–1461. 10.1093/ibd/izz046 PubMed Abstract | 10.1093/ibd/izz046 | Google Scholar 30918945 PMC6701512

[B6] Chen Y. Chiou S. Hsu A. Lin Y. Lin J. (2022). Lactobacillus rhamnosus strain LRH05 intervention ameliorated body weight gain and adipose inflammation via modulating the gut microbiota in high-fat diet-induced obese mice. Mol. Nutr. Food Res. 2021, e2100348. 10.1002/mnfr.202100348 PubMed Abstract | 10.1002/mnfr.202100348 | Google Scholar 34796638

[B7] Cho I. Yamanishi S. Cox L. Methé B. Zavadil J. Li K. (2012). Antibiotics in early life alter the murine colonic microbiome and adiposity. Nature 488, 621–626. 10.1038/nature11400 PubMed Abstract | 10.1038/nature11400 | Google Scholar 22914093 PMC3553221

[B8] Fang L. Pang Z. Shu W. Wu W. Sun M. Cong Y. (2018). Anti-TNF therapy induces CD4+ T-cell production of IL-22 and promotes epithelial repairs in patients with Crohn's disease. Inflamm. Bowel Dis. 24, 1733–1744. 10.1093/ibd/izy126 PubMed Abstract | 10.1093/ibd/izy126 | Google Scholar 29718341

[B9] Feng J. Meyer C. Wang Q. Liu J. Shirley Liu X. Zhang Y. (2012). Gfold: A generalized fold change for ranking differentially expressed genes from RNA-seq data. Bioinforma. Oxf. Engl. 28, 2782–2788. 10.1093/bioinformatics/bts515 10.1093/bioinformatics/bts515 | Google Scholar 22923299

[B10] Feuerstein J. D. Ho E. Y. Shmidt E. Singh H. Falck-Ytter Y. Sultan S. (2021). AGA clinical practice guidelines on the medical management of moderate to severe luminal and perianal fistulizing Crohn's disease. Gastroenterology 160, 2496–2508. 10.1053/j.gastro.2021.04.022 PubMed Abstract | 10.1053/j.gastro.2021.04.022 | Google Scholar 34051983 PMC8988893

[B11] Hanauer S. Sandborn W. Rutgeerts P. Fedorak R. Lukas M. MacIntosh D. (2006). Human anti-tumor necrosis factor monoclonal antibody (adalimumab) in Crohn's disease: The CLASSIC-I trial. Gastroenterology 130, 323–333. ; quiz 591. 10.1053/j.gastro.2005.11.030 PubMed Abstract | 10.1053/j.gastro.2005.11.030 | Google Scholar 16472588

[B12] Huang W. L. Wilks A. (2017). Extracellular heme uptake and the challenge of bacterial cell membranes. Annu. Rev. Biochem. 86, 799–823. 10.1146/annurev-biochem-060815-014214 PubMed Abstract | 10.1146/annurev-biochem-060815-014214 | Google Scholar 28426241

[B13] Jilani N. Akobeng A. (2007). Adalimumab for maintenance of clinical response and remission in patients with Crohn's disease: The CHARM trial. J. Pediatr. Gastroenterol. Nutr. 132, 52–65. Colombel JF, Sandborn WJ, Rutgeerts P et al. Gastroenterology 2007;132:52-65. 2008;46:226-7. 10.1097/MPG.0b013e318156e139 10.1097/MPG.0b013e318156e139 | Google Scholar 17241859

[B14] Kostic A. Xavier R. Gevers D. (2014). The microbiome in inflammatory bowel disease: Current status and the future ahead. Gastroenterology 146, 1489–1499. 10.1053/j.gastro.2014.02.009 PubMed Abstract | 10.1053/j.gastro.2014.02.009 | Google Scholar 24560869 PMC4034132

[B15] Kramer J. Oezkaya O. Kuemmerli R. (2020). Bacterial siderophores in community and host interactions. Nat. Rev. Microbiol. 18, 152–163. 10.1038/s41579-019-0284-4 PubMed Abstract | 10.1038/s41579-019-0284-4 | Google Scholar 31748738 PMC7116523

[B16] Meehan C. Beiko R. (2014). A phylogenomic view of ecological specialization in the Lachnospiraceae, a family of digestive tract-associated bacteria. Genome Biol. Evol. 6, 703–713. 10.1093/gbe/evu050 PubMed Abstract | 10.1093/gbe/evu050 | Google Scholar 24625961 PMC3971600

[B17] Mo L. Zhao G. Li X. He N. Xiao X. Xu H. (2021). Biocatalytical acyl-modification of puerarin: Shape gut microbiota profile and improve short chain fatty acids production in rats. Dordrecht, Netherlands: Plant foods for human nutrition. Google Scholar 10.1007/s11130-021-00936-1 34822099

[B18] Papamichael K. Lin S. Moore M. Papaioannou G. Sattler L. Cheifetz A. (2019). Infliximab in inflammatory bowel disease. Ther. Adv. Chronic Dis. 10, 2040622319838443. 10.1177/2040622319838443 PubMed Abstract | 10.1177/2040622319838443 | Google Scholar 30937157 PMC6435871

[B19] Rajca S. Grondin V. Louis E. Vernier-Massouille G. Grimaud J. Bouhnik Y. (2014). Alterations in the intestinal microbiome (dysbiosis) as a predictor of relapse after infliximab withdrawal in Crohn's disease. Inflamm. Bowel Dis. 20, 978–986. 10.1097/MIB.0000000000000036 PubMed Abstract | 10.1097/MIB.0000000000000036 | Google Scholar 24788220

[B20] Rutgeerts P. Goboes K. Peeters M. Hiele M. Penninckx F. Aerts R. (1991). Effect of faecal stream diversion on recurrence of Crohn's disease in the neoterminal ileum. Lancet 338, 771–774. 10.1016/0140-6736(91)90663-a PubMed Abstract | 10.1016/0140-6736(91)90663-a | Google Scholar 1681159

[B21] Sartor R. B. (2004). Therapeutic manipulation of the enteric microflora in inflammatory bowel diseases: Antibiotics, probiotics, and prebiotics. Gastroenterology 126, 1620–1633. 10.1053/j.gastro.2004.03.024 PubMed Abstract | 10.1053/j.gastro.2004.03.024 | Google Scholar 15168372

[B22] Satsangi J. Silverberg M. S. Vermeire S. Colombel J. F. (2006). The montreal classification of inflammatory bowel disease: Controversies, consensus, and implications. Gut 55, 749–753. 10.1136/gut.2005.082909 PubMed Abstract | 10.1136/gut.2005.082909 | Google Scholar 16698746 PMC1856208

[B23] Scott C. Arora G. Dickson K. Lehmann C. (2021). Iron chelation in local infection. Molecules 26, E189. 10.3390/molecules26010189 PubMed Abstract | 10.3390/molecules26010189 | Google Scholar 33401708 PMC7794793

[B24] Seyoum Y. Baye K. Humblot C. (2021). Iron homeostasis in host and gut bacteria - a complex interrelationship. Gut Microbes 13, 1–19. 10.1080/19490976.2021.1874855 10.1080/19490976.2021.1874855 | Google Scholar PMC7872071 33541211

[B25] Vuitton L. Marteau P. Sandborn W. J. Levesque B. G. Feagan B. Vermeire S. (2016). IOIBD technical review on endoscopic indices for Crohn's disease clinical trials. Gut 65, 1447–1455. 10.1136/gutjnl-2015-309903 PubMed Abstract | 10.1136/gutjnl-2015-309903 | Google Scholar 26353983

[B26] Wang Y. Gao X. Ghozlane A. Hu H. Li X. Xiao Y. (2018). Characteristics of faecal microbiota in paediatric Crohn's disease and their dynamic changes during infliximab therapy. J. Crohns Colitis 12, 337–346. 10.1093/ecco-jcc/jjx153 PubMed Abstract | 10.1093/ecco-jcc/jjx153 | Google Scholar 29194468

[B27] Xu L. Dong Z. B. Chiniquy D. Pierroz G. Deng S. W. Gao C. (2021). Genome-resolved metagenomics reveals role of iron metabolism in drought-induced rhizosphere microbiome dynamics. Nat. Commun. 12, 3209. 10.1038/s41467-021-23553-7 PubMed Abstract | 10.1038/s41467-021-23553-7 | Google Scholar 34050180 PMC8163885

[B28] Zhuang X. Tian Z. Feng R. Li M. Li T. Zhou G. (2020). Fecal microbiota alterations associated with clinical and endoscopic response to infliximab therapy in Crohn's disease. Inflamm. Bowel Dis. 26, 1636–1647. 10.1093/ibd/izaa253 PubMed Abstract | 10.1093/ibd/izaa253 | Google Scholar 33026078

