# Supplementary figures and images for: Distinct alterations of fecal microbiota refer to the efficacy of adalimumab in Crohn’s disease
Source: Front Pharmacol. 2022 Aug 11;13:913720. doi: 10.3389/fphar.2022.913720 (PMC9410713; doi:10.3389/fphar.2022.913720)

## Slide 1
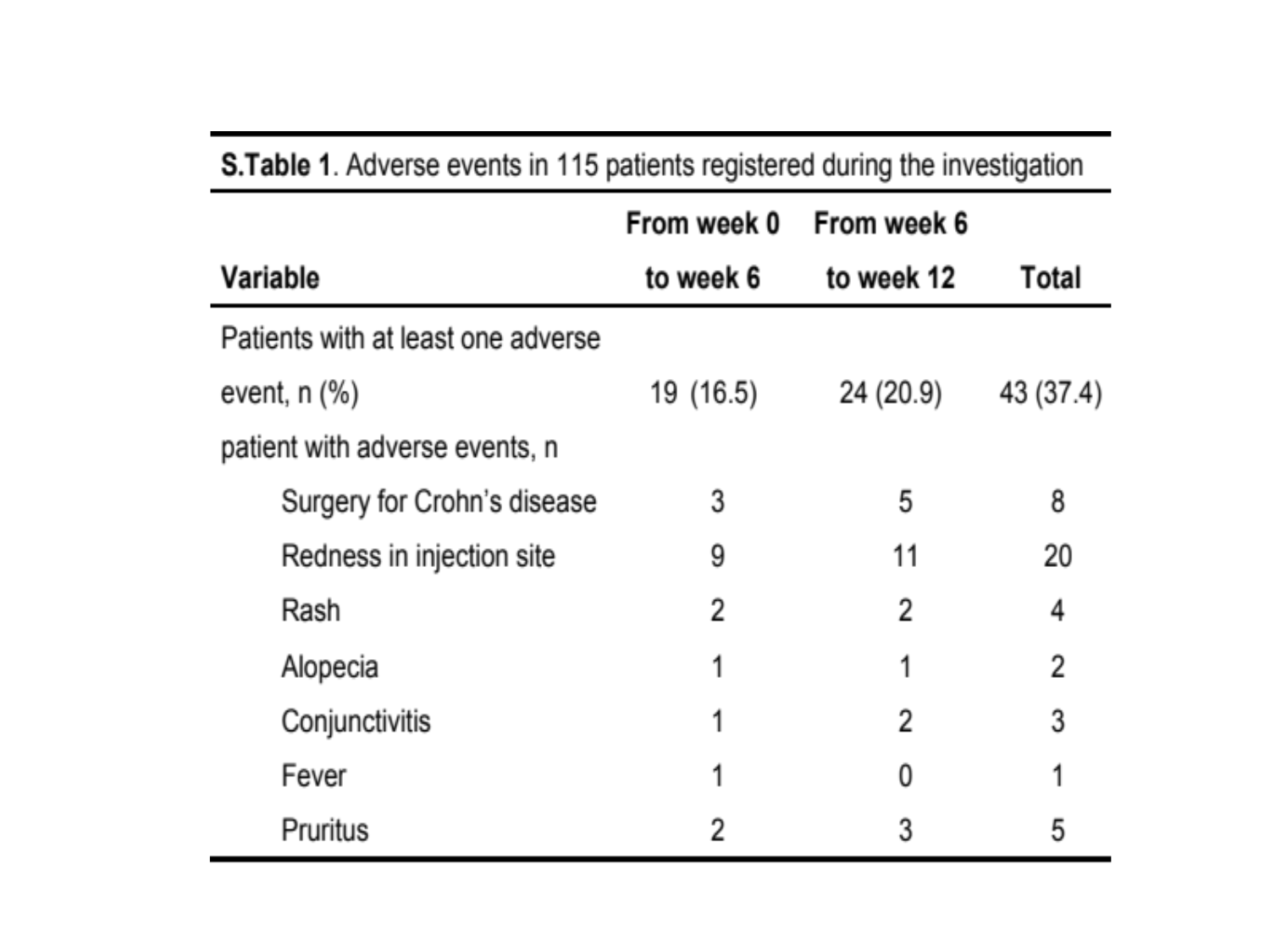

## Slide 2
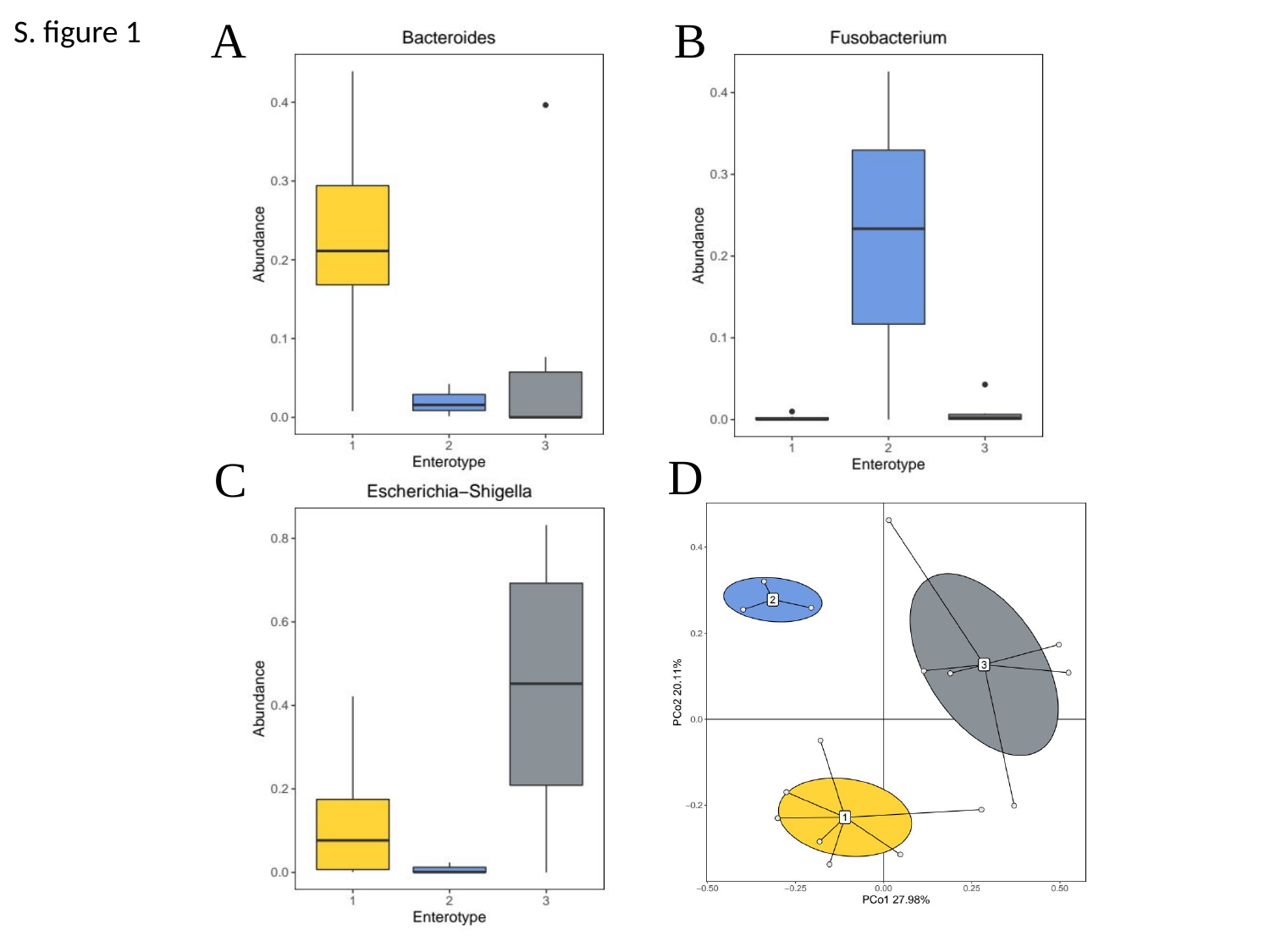

A
B
S. figure 1
D
C

Supplement: Supplementary file 1 [file Presentation1.pptx]
